# ProbioMinServer: an integrated platform for assessing the safety and functional properties of potential probiotic strains

**DOI:** 10.1093/bioadv/vbad153

**Published:** 2023-10-18

**Authors:** Yen-Yi Liu, Chu-Yi Hsu, Ya-Chu Yang, Chien-Hsun Huang, Chih-Chieh Chen

**Affiliations:** Department of Biology, National Changhua University of Education, Changhua 500207, Taiwan; Institute of Medical Science and Technology, National Sun Yat-sen University, Kaohsiung 804201, Taiwan; Institute of Medical Science and Technology, National Sun Yat-sen University, Kaohsiung 804201, Taiwan; Bioresource Collection and Research Center, Food Industry Research and Development Institute, Hsinchu 300193, Taiwan; Institute of Medical Science and Technology, National Sun Yat-sen University, Kaohsiung 804201, Taiwan

## Abstract

**Motivation:**

ProbioMinServer is a platform designed to help researchers access information on probiotics regarding a wide variety of characteristics, such as safety (e.g. antimicrobial resistance, virulence, pathogenic, plasmid, and prophage genes) and functionality (e.g. functional classes, carbohydrate-active enzyme, and metabolite gene cluster profile). Because probiotics are functional foods, their safety and functionality are a crucial part of health care. Genomics has become a crucial methodology for investigating the safety and functionality of probiotics in food and feed. This shift is primarily attributed to the growing affordability of next-generation sequencing technologies. However, no integrated platform is available for simultaneously evaluating probiotic strain safety, investigating probiotic functionality, and identifying known phylogenetically related strains.

**Results:**

Thus, we constructed a new platform, ProbioMinServer, which incorporates these functions. ProbioMinServer accepts whole-genome sequence files in the FASTA format. If the query genome belongs to the 25 common probiotic species collected in our database, the server performs a database search and analyzes the core-genome multilocus sequence typing. Front-end applications were implemented in JavaScript with a bootstrap framework, and back-end programs were implemented using PHP, Perl, and Python. ProbioMinServer can help researchers quickly and easily retrieve information on the safety and functionality of various probiotics.

**Availability and implementation:**

The platform is available at https://probiomindb.imst.nsysu.edu.tw.

## 1 Introduction

Probiotics are defined by the Food and Agriculture Organization of the United Nations and the WHO as “live microorganisms which, when administered in adequate amounts, confer a health benefit on the host” (Food and Agriculture Organization of the United Nations 2001). They can be administered either as a single or mixed live bacterial strain. Bacterial probiotic strains are commonly from the genera *Bacillus*, *Bifidobacterium*, *Lactobacillus*, and *Streptococcus* (Holzapfel *et al.* 2001, Fijan 2014). Because next-generation sequencing is gradually becoming less expensive, whole-genome-sequence (WGS)-based probiotic genome comparison has emerged as the predominant method for analyzing food and feed safety (Koirala and Anal 2021, Syromyatnikov *et al.* 2022). Consequently, researchers have an increasing demand for a user-friendly platform that facilitates comprehensive whole-genome analyses of probiotics.

The functional properties of probiotics include the regulation of genes or metabolic pathways that are responsible for the production of vitamins (Rossi *et al.* 2011, Capozzi *et al.* 2012), synthesis of essential amino acids (Portune *et al.* 2016, Jäger *et al.* 2020), generation of antioxidants (Wang *et al.* 2017), digestion of complex carbohydrates (Flint *et al.* 2012), mitigation of antibiotic side effects (Zhou *et al.* 2005), and modulation of the immune system (Servin 2004, Klaenhammer *et al.* 2012). Probiotic strains should be free of virulence factors (VFs) and have no multidrug-resistant properties. In recent years, probiotic safety has become increasingly important globally, especially after the discovery of vancomycin-resistant *Enterococcus* strains (Cetinkaya *et al.* 2000, Vidal *et al.* 2010). Because these vancomycin-resistant genes can be transferred to other enterococci species and spread drug resistance, the WHO listed vancomycin-resistant *Enterococcus* as a high-threat pathogen in early 2017 (Tacconelli *et al.* 2018). This drew the attention of researchers to the risk of horizontal transfer of antibiotic genes (Li *et al.* 2020, Tóth *et al.* 2021) and VFs (Ghattargi *et al.* 2018) among probiotic bacteria. In addition to gene acquisition, genes can also undergo duplication or loss, which makes interpretation of patterns of genetic diversity more challenging (Makarova and Koonin 2007). Consequently, it is crucial for researchers to identify antimicrobial resistance and virulence genes and evaluate horizontal gene transfer capability to assess the safety of probiotics.

Whole-genome analysis is a standard method for assessing the safety of probiotics (Wang *et al.* 2021). A live platform called iProbiotics (Sun *et al.* 2022) was designed to predict the probiotic probability of a bacterial strain with whole-genome primary sequences; however, no platform provides integrated services for evaluating the safety and functional properties of a potential probiotic strain. Such a platform would have both academic and industrial applications because it would allow researchers to rapidly perform species identification, genome-based safety assessment, and functional annotation. This could help researchers evaluate the safety and functional properties of their studied bacteria more efficiently.

We developed ProbioMinServer, an integrated platform that can be used to analyze the safety and functionality of bacterial genomes and evaluate probiotic potential. The platform compares the WGS of uploaded strains with built-in databases to analyze the antibiotic resistance genes (ARGs), VFs, pathogenic genes (PGs), plasmid types, prophage regions, functional annotation, carbohydrate-active enzyme (CAZy), and metabolite gene cluster profile. When the query genome corresponds to any of the 25 common probiotic species stored in our ProbioMinServer, core-genome multilocus sequence typing (cgMLST) facilitates the presentation of the most closely phylogenetically related probiotic isolates. Users can easily interface with the built-in databases through keyword searches, browsing, and downloads. We believe that ProbioMinServer is highly useful for probiotic development.

## 2 Methods

**Figure 1.**
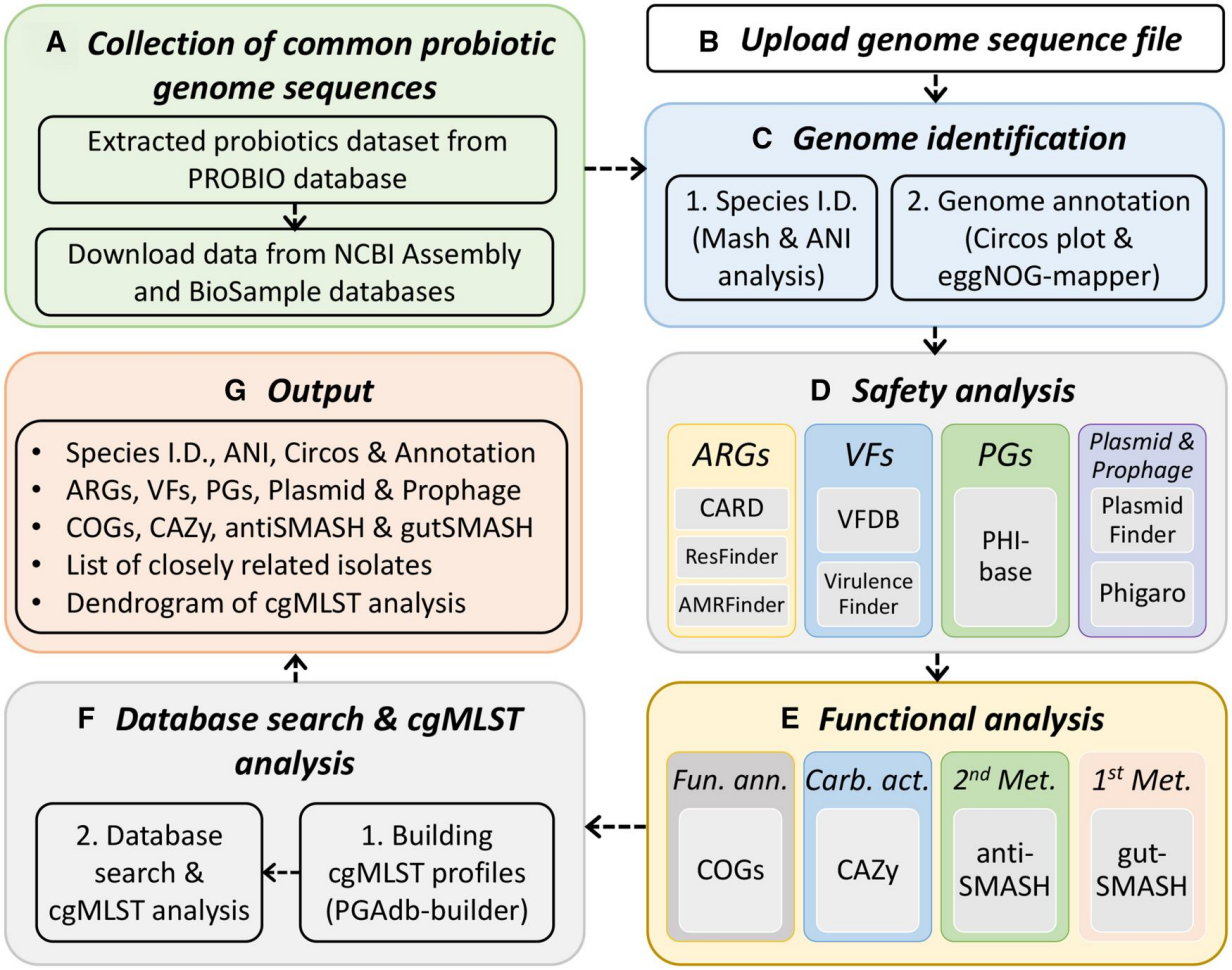
Workflow of ProbioMinServer.

Figure 1 illustrates the workflow of ProbioMinServer, which contains information on the 25 most common species of probiotics (Fig. 1A). This information can be used to mine information on the characteristics of probiotics, such as their safety and functional properties. Users can upload a query genome sequence or enter a National Center for Biotechnology Information (NCBI) Assembly ID (Fig. 1B). The server first performs *in silico* genome identification, which includes species identification and genome annotation (Fig. 1C). Subsequently, safety (Fig. 1D) and functional (Fig. 1E) analysis is performed using state-of-the-art software to detect ARGs, VFs, PGs, plasmids, and prophages; classify data on Clusters of Orthologous Groups of proteins (COGs) functions and CAZys; and analyze metabolic pathways. If the analyzed strain is belonging to the 25 common probiotic species collected in our database, the number of allelic differences and cgMLST profiles are used to calculate the genetic distance and evaluate the phylogenetic relationship of the strains (Fig. 1F). Supplementary Table S1 lists the software and databases used in ProbioMinServer. JavaScript libraries are used for visualizing results (Fig. 1G). cgMLST analysis was built on the basis of our PGAdb-builder (Liu *et al.* 2016, 2019). Python and Perl scripts are used to integrate the analytic pipeline. The web page was constructed using HTML and PHP, and the web server runs on a Linux system powered by 48-core Intel Xeon CPUs clocked at 2.30 GHz with 128 GB RAM.

### 2.1 WGSs of common probiotic species

ProbioMinServer covers 25 common probiotic species (belonging to 11 genera) whose data were extracted from the PROBIO database (Tao *et al.* 2017) through bacterial species and strain name matching using the corresponding scientific name or NCBI taxonomic identifier. The genome sequences were downloaded from the NCBI Assembly database (Fig. 1A). To collect high-quality genome sequences, we applied the following filters to the NCBI Assembly database: “latest version file,” “exclude anomalous,” and “taxonomy check.” Table 1 lists the genus, species names, and number of isolates included in ProbioMinServer. Each genome entry was mapped to the NCBI BioSample database to obtain information on biological source materials used in experimental assays, such as identifiers, strain names, and hosts.

**Table 1.** Names of genera and species and number of isolates in ProbioMinServer.

Genus	Species	Number of isolates
*Alkalihalobacillus*	*Alkalihalobacillus clausii*	37
*Bacillus*	*Bacillus amyloliquefaciens*	138
	*Bacillus subtilis*	436
*Bifidobacterium*	*Bifidobacterium animalis* subsp. *lactis*	57
	*Bifidobacterium bifidum*	134
	*Bifidobacterium breve*	168
	*Bifidobacterium longum* subsp. *infantis*	48
	*Bifidobacterium pseudocatenulatum*	167
	*Bifidobacterium thermophilum*	8
*Lacticaseibacillus*	*Lacticaseibacillus paracasei*	188
	*Lacticaseibacillus rhamnosus*	240
*Lactiplantibacillus*	*Lactiplantibacillus plantarum*	652
*Lactobacillus*	*Lactobacillus acidophilus*	65
	*Lactobacillus crispatus*	316
	*Lactobacillus delbrueckii* subsp. *bulgaricus*	52
	*Lactobacillus gasseri*	76
	*Lactobacillus helveticus*	161
	*Lactobacillus johnsonii*	78
*Levilactobacillus*	*Levilactobacillus brevis*	101
*Ligilactobacillus*	*Ligilactobacillus salivarius*	205
*Limosilactobacillus*	*Limosilactobacillus fermentum*	114
	*Limosilactobacillus reuteri*	331
*Streptococcus*	*Streptococcus salivarius*	206
	*Streptococcus thermophilus*	184
*Weizmannia*	*Weizmannia coagulans*	48
Total		4210

### 2.2 Genome identification

To identify the user-uploaded genome sequence (Fig. 1B), ProbioMinServer first performs *in silico* genome identification using Mash v2.3 (Ondov *et al.* 2016) to search against NCBI prokaryote type strains (August 2023 release) and then average nucleotide identity (ANI) analysis (Richter and Rossello-Mora 2009) is provided to calculate the genome similarity between the query strain and the subject type strains. The ANI cutoff of 96% is applied for species identification. Mummer2circos v1.4.2 (Kurtz *et al.* 2004) and Circos v0.69–8 (Krzywinski *et al.* 2009) are used to align and plot the query strain against the subject type strain. EggNOG-mapper v2 (Cantalapiedra *et al.* 2021), a tool for fast functional annotation of novel sequences, is then used for genome annotation and ortholog assignment (Fig. 1C).

### 2.3 Safety and functional analysis

Information on the safety and functional properties of genomes are provided by a variety of software. For safety analysis, Resistance Gene Identifier v6.0.0 software (BLAST homolog detection) searches against the Comprehensive Antibiotic Resistance Database (CARD) Variants v4.0.0 (McArthur *et al.* 2013), ResFinder v4.0 (Bortolaia *et al.* 2020), and AMRFinderPlus v3.10.42 (Feldgarden *et al.* 2021) are used to detect ARGs. BLASTN v2.8.1+ searches against the setB database of the pathogenic Virulence Factor Database (VFDB) (June 2022 release) (Chen *et al.* 2005) and VirulenceFinder v2.0.3 (Malberg Tetzschner *et al.* 2020) is used to detect VFs. Most of the selected tools are suggested in the European Food Safety Authority (EFSA) guidance (EFSA FEEDAP 2018) and widely used for microbial safety assessment. To obtain the pathogenicity of the query genome, BLASTP v2.8.1+ searches are conducted against the Pathogen Host Interactions database v4.14 (Urban *et al.* 2020) to identify potential PGs. To detect the mobile genetic elements of the query genome, PlasmidFinder v2.0.1 (Carattoli *et al.* 2014) and Phigaro v2.3.0 (Starikova *et al.* 2020) are used for the identification of plasmid replicons and typing of prophage regions in the genome sequence, respectively. Based on EFSA statement (EFSA 2021), these results are filtered using parameters with ≥80% identity and ≥70% coverage for gene identification (Fig. 1D). In addition to conducting descriptive analysis, we also computed the probiotic potential risk index and probiotic potential risk score (PPRS) as defined by Bai *et al.* (2022) to evaluate the risks associated with each probiotic. The score was classified as low-risk (≤4), medium-risk (4–6), and high-risk (≥6). For functional analysis, the COGs (Tatusov *et al.* 2001) and CAZy (Lombard *et al.* 2014) databases are used for functional annotation and classification of CAZys. Furthermore, antiSMASH v6.0.0 (Medema *et al.* 2011) and gutSMASH v1.0.0 (Pascal Andreu *et al.* 2021) are used with default parameters to detect potential secondary and primary metabolite biosynthesis gene clusters, since the encoded bioactive secondary metabolites can play important roles in microbe–microbe and host–microbe interactions (Fig. 1E).

### 2.4 Database search and cgMLST

If the analyzed strain is belonging to the 25 common probiotic species collected in our database, ProbioMinServer performs database searches (Fig. 1F) and cgMLST analysis to obtain a high-resolution tree of the phylogenetic relatedness of the top-20 isolates with the smallest allelic distances (Fig. 1G). We used PGAdb-builder to construct the cgMLST scheme, core-genome database, and allelic profiling (Liu *et al.* 2016, 2019). We also used the clustering algorithm of the neighbor-joining method in PHYLIP v3.6 to construct the genetic relatedness tree from the established allelic sequence (Felsenstein 1981). The ETE3 v3.1.1 toolkit was used to calculate bootstrap values in a dendrogram (Huerta-Cepas *et al.* 2016).

## 3 Web server

### 3.1 Input format

ProbioMinServer accepts two types of inputs: a genome sequence file in FASTA format or an NCBI Assembly ID. For a genome sequence with a size of ∼5 MB, species identification, genome annotation, safety and functional analysis, database search, and cgMLST analysis require ∼30 min. However, the run time can exceed 1 h with longer sequences. Therefore, users are encouraged to enter their email address, to which a notification is sent when the analysis is complete.

### 3.2 Output format

**Figure 2.**
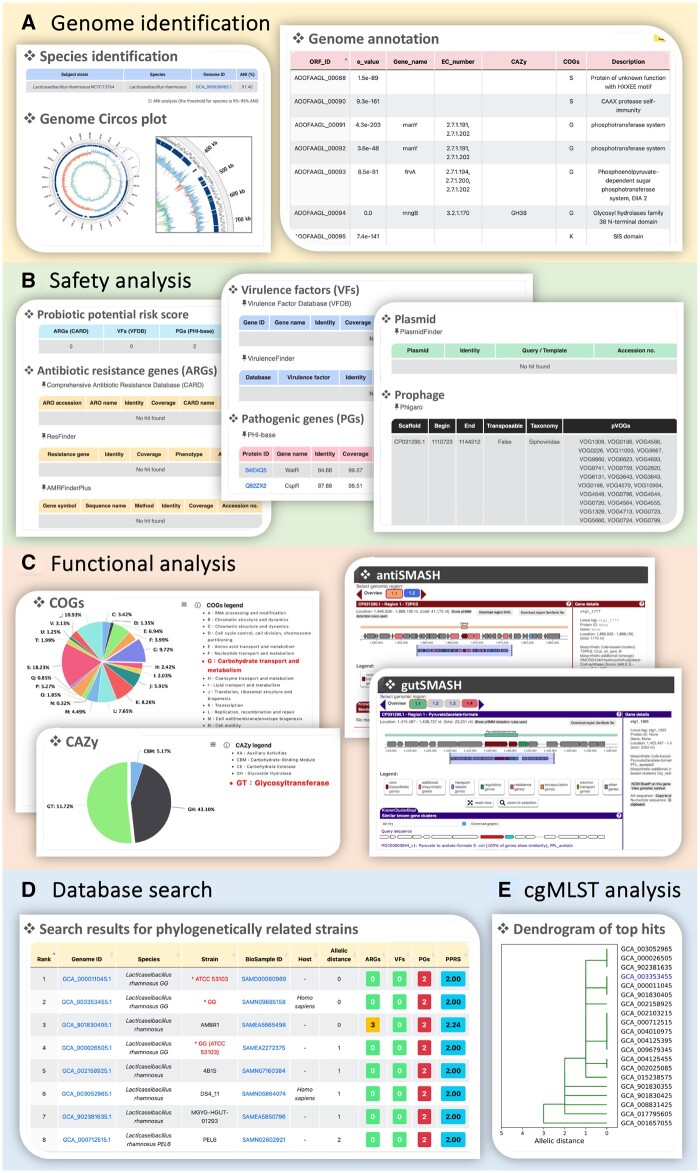
Features of ProbioMinServer. (A) Output page of genome identification. (B) Results of safety analysis. (C) Results of functional analysis. (D) Results of database search. (E) Results of cgMLST analysis.

ProbioMinServer presents the results in 3–5 tabs, depending on the species of the query genome (Fig. 2). The first tab provides species identification, a genome Circos plot, and genome annotation (Fig. 2A). The query strain (light gray) is aligned to the subject type strain (dark blue) obtained from the NCBI Genome database. The GC skew (blue/red) and GC content/variation (blue/green) are also presented in the Circos plot. The second tab provides the results of safety analysis include tables listing the ARGs, VFs, PGs, and plasmid and prophage genes, which are all detected with various methods (Fig. 2B). The third tab provides the results of the functional analysis include pie charts representing the distributions of COG functional annotations and CAZy classifications, and tables listing the secondary and primary metabolite gene clusters (Fig. 2C). In the case that the query genome belongs to one of the probiotic species listed in the database, an additional two tabs will be presented. The fourth tab provides the results of the database search include a table listing the top 100 isolates with the smallest allelic distances based on the cgMLST analysis (Fig. 2D). Each record includes the genome ID; species name; strain name; BioSample ID; host; allelic distance; number of ARGs, VFs, and PGs; and the value of PPRS. Detailed information is presented when the user clicks on the icon corresponding to the genome ID’s. Data can be sorted by clicking on a column header, and the data can be downloaded as a tab-delimited file. If the isolate has been annotated as a probiotic in the literature, the strain name is preceded by an asterisk and is colored red, enabling users to conduct further investigation. Finally, in the fifth tab, the results of the phylogenetic relatedness tree analysis performed using cgMLST are presented (Fig. 2E), and the server allows users to download the phylogenetic relatedness tree in Newick and PDF formats.

### 3.3 Browse

The browse module is designed to present visualizations of the analysis results and the genetic relationships among isolates deposited in ProbioMinServer. Each “browse” page lists a summary of each of the isolates in a specific species. Strains annotated as probiotics in the literature are indicated with an asterisk and are colored red. The user can access detailed information on a specific isolate by clicking on the genome ID.

## 4 Example analysis

**Figure 3.**
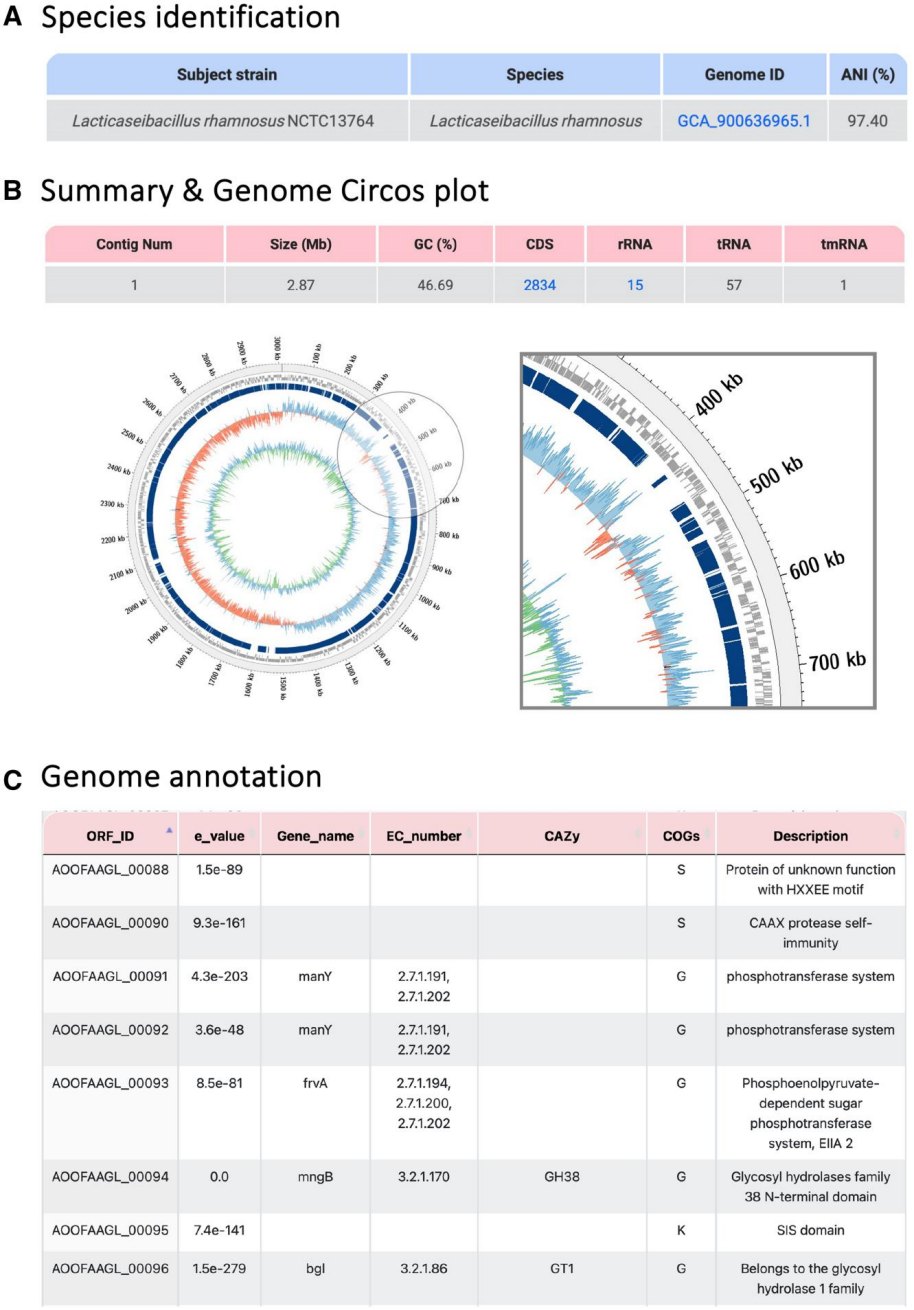
Genome identification of *L.rhamnosus* GG. (A) Species identification. (B) Summary and Genome Circos plot used to compare the query strain (light gray) with subject type strain (dark blue), GC skew (blue/red), and GC content/variation (blue/green). (C) Results of genome annotation.

To demonstrate the effectiveness of ProbioMinServer in safety and functionality analyses of potential probiotic strains, a genome sequence from *Lacticaseibacillus rhamnosus* Gorbach–Goldin (GG) (NCBI Assembly ID: GCA_003353455.1) was used as the query in an example analysis. The strain was isolated from *Homo sapiens* in 2016 in South Korea. ProbioMinServer first identified this strain as *L.rhamnosus* and the ANI analysis showed a high ANI value (97.40%) with the type strain *L.rhamnosus* NCTC13764 (Fig. 3A). The results of the basic analyses for the uploaded GCA_003353455.1 were as follows: contig number: 1; genome size: 2.87 Mb; GC content: 46.69%; and number of annotations in CDS, rRNA, tRNA, and tmRNA: 2834, 15, 57, and 1, respectively. The query genome sequence was aligned with the type strain to generate a genome Circos plot (Fig. 3B). This plot indicated no large differences in the query genome (light gray), and only some small gaps were observed relative to the type strain (dark blue). We also presented further annotations on, e.g. CAZy, COGs, and enzyme classification number for each of the open reading frames in the genome (Fig. 3C).

In a safety analysis, we employed three methods for ARG detection; two methods for VF detection; and one method for detecting PGs, plasmids, and prophages. The analytical results on GCA_003353455.1 revealed no ARGs, VFs, or plasmids; two PGs; three prophage regions; and the PPRS is 2.00 (Supplementary Fig. S1). Supplementary Figure S2A indicates the results of the functional analysis of COGs, with carbohydrate transport and metabolism being the most frequently observed orthologous group. Results with “function unknown” and “unclassified” were excluded. CAZy analysis indicated that glycosyltransferase and glycoside hydrolase were the most abundant enzymes in GCA_003353455.1 (Supplementary Fig. S2B). Both COGs and CAZy analysis inferred the probiotic potential of the query isolate. antiSMASH (Supplementary Fig. S3A) and gutSMASH (Supplementary Fig. S3B) indicated the secondary and primary metabolites, which included T3PKS, RiPP-like, Pyruvate to acetate-formate, and Gallic acid metabolism gene clusters. In previous studies, T3PKS and RiPP-like gene clusters were identified in *Pseudovibrio* and *Lactiplantibacillus plantarum* 13–3, which indicated their potential use in food processing due to their production of novel bioactive compounds (Naughton *et al.* 2017, Aziz *et al.* 2022).

**Figure 4.**
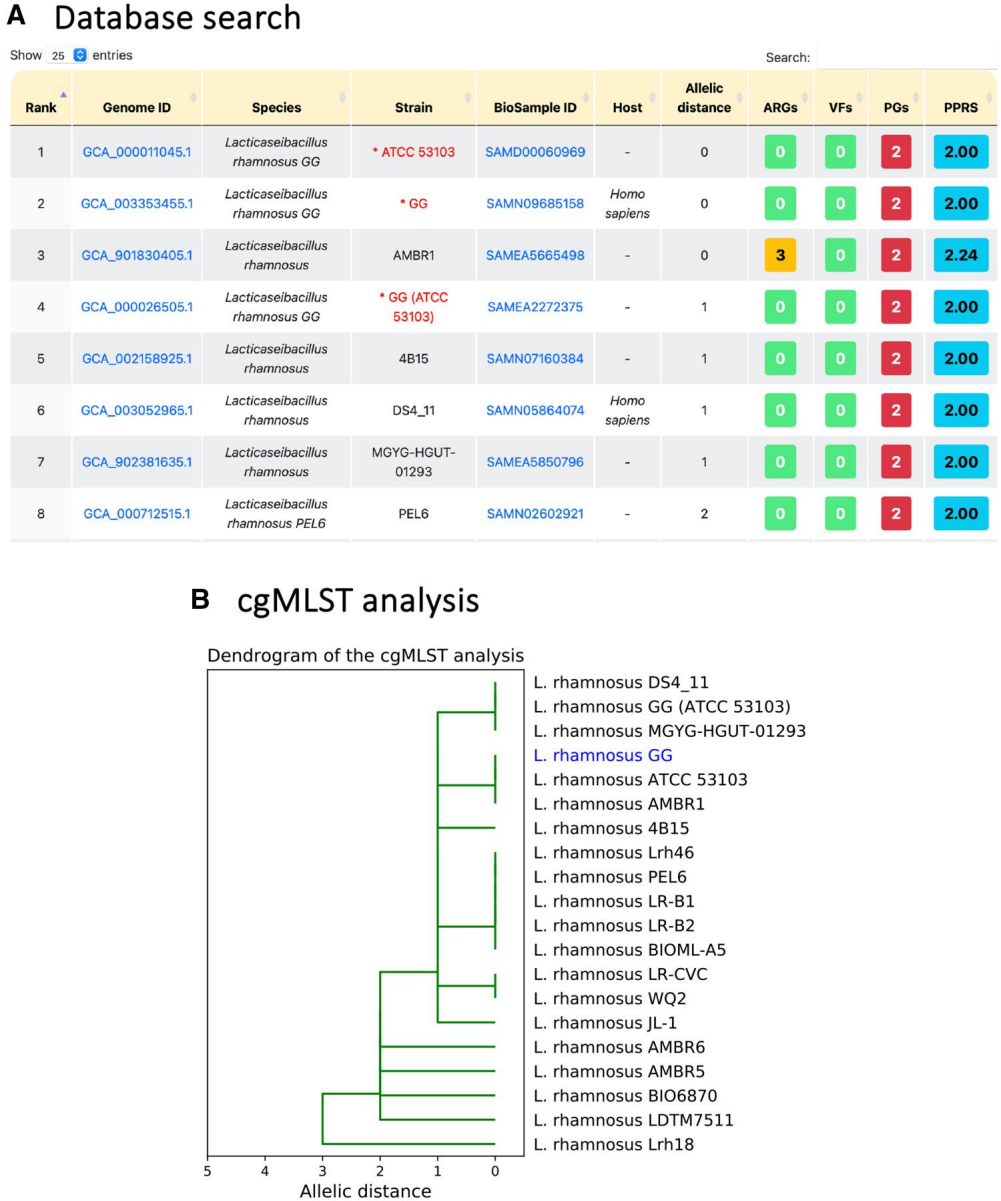
Database search and cgMLST analysis of *L.rhamnosus* GG. (A) Results of database search. Each record includes the genome ID; species name; strain name; BioSample ID; host; allelic distance; number of ARGs, VFs, and PGs; and the value of PPRS. Strains annotated as probiotics are indicated with an asterisk and are colored red. (B) Dendrogram of the top 20 hit strains with the smallest allelic distances.

The results of the database search for GCA_003353455.1 using the cgMLST methodology are presented in Fig. 4A and Supplementary Table S2. These results include information on, e.g. the genome ID; species name; strain name; BioSample ID; host; allelic distance; number of ARGs, VFs, and PGs; and the value of PPRS. Figure 4B indicates that the strains ATCC 53103 and AMBR1 have zero allelic distance compared with the query isolate GG (GCA_003353455.1). However, AMBR1 contains more ARGs. This may explain why ATCC 53103 and the query isolate were selected as potential probiotics. The combined safety and functionality analyses conducted on the query isolate *L.rhamnosus* GG using ProbioMinServer indicated its probiotic potential.

This implementation of ProbioMinServer demonstrated its ability to provide comprehensive information regarding the safety and functionality of the query genome and list the isolates (25 probiotic species listed in the database) with similar cgMLST profiles. Nevertheless, it should be noted that our assessment method can only provide guidance for the selection of probiotics. If necessary, more experimental techniques should be used to validate the selected probiotics.

## 5 Conclusion

ProbioMinServer mines the probiotic potential of query genomes with safety and functionality analyses. Conducting cgMLST-based extensive comparison with database of probiotic bacterial species helps users find strains similar to the query isolate. To our knowledge, ProbioMinServer is the first integrated web server that comprehensively mines information on probiotic potential from a bacterial WGS. One limitation of ProbioMinServer is that the database search and cgMLST analysis are only performed on query genomes belonging to the 25 common probiotic species collected in our database. The example analysis demonstrated that ProbioMinServer is effective in performing safety and functionality analyses and extensive comparisons with the integrated probiotic database. It facilitates thorough investigations into the probiotic potential of query isolates. ProbioMinServer can assist scientists in conducting safety and functionality assessments and evaluating the probiotic potential of bacterial strains for development of probiotic products.

## Supplementary Material

vbad153_Supplementary_Data

## Data Availability

ProbioMinServer is freely available at https://probiomindb.imst.nsysu.edu.tw.
