# Supplementary material for: ProbioMinServer: an integrated platform for assessing the safety and functional properties of potential probiotic strains
Source: Bioinform Adv. 2023 Oct 18;3(1):vbad153. doi: 10.1093/bioadv/vbad153 (PMC10625473; doi:10.1093/bioadv/vbad153)
Supplement: vbad153_Supplementary_Data [file vbad153_supplementary_data.pdf]

ProbioMinServer: an integrated platform for assessing the safety and functional properties of potential probiotic strains

Yen-Yi Liu[1], Chu-Yi Hsu[2], Ya-Chu Yang[2], Chien-Hsun Huang[3], Chih-Chieh Chen[2]*

[1]*Department of Biology, National Changhua University of Education, Changhua, Taiwan*
[2]*Institute of Medical Science and Technology, National Sun Yat-sen University, Kaohsiung, Taiwan*
[3]*Bioresource Collection and Research Center, Food Industry Research and Development Institute, Hsinchu, Taiwan*

Keywords: probiotics; probiotic safety and functionality assessment; core-genome multilocus sequence typing (cgMLST); visualized database platform

*Correspond to: Chih-Chieh Chen, Institute of Medical Science and Technology, National Sun Yat-sen University, Kaohsiung, Taiwan. Phone: +886-7-5252000 ext. 7156. E-mail: chieh@imst.nsysu.edu.tw

Email addresses:

YYL: current7888@gmail.com
CYH: julie41020@gmail.com
YCY: karta101313@gmail.com
CHH: chh@firdi.org.tw
CCC: chieh@imst.nsysu.edu.tw

(A) Probiotic potential risk scores

ARGs (CARD)	VFs (VFDB)	PGs (PHI-base)	PPRS
0	0	2	2.00

(B) Antibiotic resistance genes

Comprehensive Antibiotic Resistance Database (CARD)

ARO accession	ARO name	Identity	Coverage	CARD name	Accession no.
No hit found					

ResFinder

Resistance gene	Identity	Coverage	Phenotype	Accession no.
No hit found				

AMRFinderPlus

Gene symbol	Sequence name	Method	Identity	Coverage	Accession no.
No hit found					

(C) Virulence factors

Virulence Factor Database (VFDB)

Gene ID	Gene name	Identity	Coverage	e-value	VF name	VF category	Accession no.
No hit found							

VirulenceFinder

Database	Virulence factor	Identity	Coverage	Protein function	Accession no.
No hit found					

(D) Pathogenic genes

PHI-base

Protein ID	Gene name	Identity	Coverage	e-value	Function	Gene ID
S4E4Q5	WalR	84.68	99.57	1.80e-144	Transcriptional regulatory protein	EPH95667
Q82ZX2	CspR	87.88	98.51	1.62e-40	Cold shock protein	AAO82613

(E) Plasmid

PlasmidFinder

Plasmid	Identity	Query / Template	Accession no.
No hit found			

(F) Prophage

Phigaro

Scaffold	Begin	End	Transposable	Taxonomy	pVOGs
CP031290.1	1110723	1144312	False	Siphoviridae	VOG1309, VOG0186, VOG4566, VOG0226, VOG11003, VOG9667, VOG9860, VOG6623, VOG4693, VOG9741, VOG9759, VOG2820, VOG6131, VOG3643, VOG3643, VOG0198, VOG4570, VOG10904, VOG4549, VOG0796, VOG4544, VOG0720, VOG4564, VOG4555, VOG1329, VOG4713, VOG0723, VOG5660, VOG0724, VOG0799, VOG0725, VOG0800, VOG4545, VOG4605, VOG4599, VOG6455, VOG7896, VOG1637, VOG0054, VOG4918
CP031290.1	1528633	1552537	False	Siphoviridae	VOG0275, VOG6495, VOG8294, VOG0703, VOG0753, VOG7896, VOG6455, VOG4599, VOG4605, VOG6163, VOG0660, VOG0209, VOG4586, VOG0207, VOG0205, VOG4568, VOG4556, VOG0202, VOG1886, VOG4581
CP031290.1	2951627	2964261	False	Siphoviridae	VOG1581, VOG4632, VOG4609, VOG4841, VOG4581, VOG4544, VOG5051, VOG4556, VOG4568, VOG0204, VOG4589

Figure S1. Safety analysis of *Lactobacillus rhamnosus* GG

(A) The results of PPRS. Detection of (B) the ARGs based on CARD search, ResFinder, and AMRFinderPlus; (C) VFs based on VFDB search and VirulenceFinder; (D) PGs based on PHI-base search; (E) plasmid appearance and type based on PlasmidFinder; and (F) the prophage regions based on Phigaro

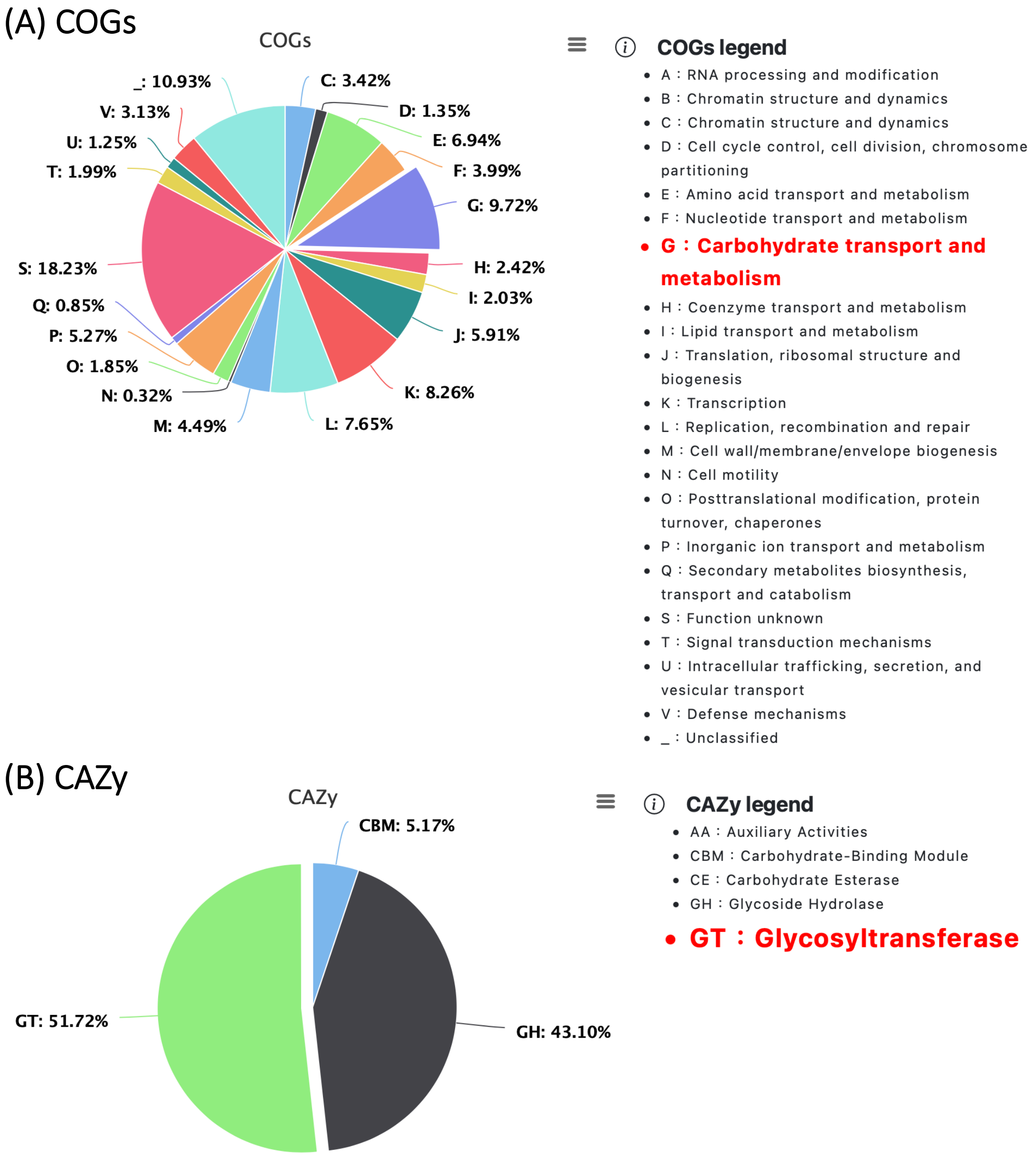

Figure S2. Functional analysis of *Lactobacillus rhamnosus* GG

Distributions of (A) COGs functional annotations and (B) carbohydrate-active enzymes (CAZy) classifications

(A) antiSMASH

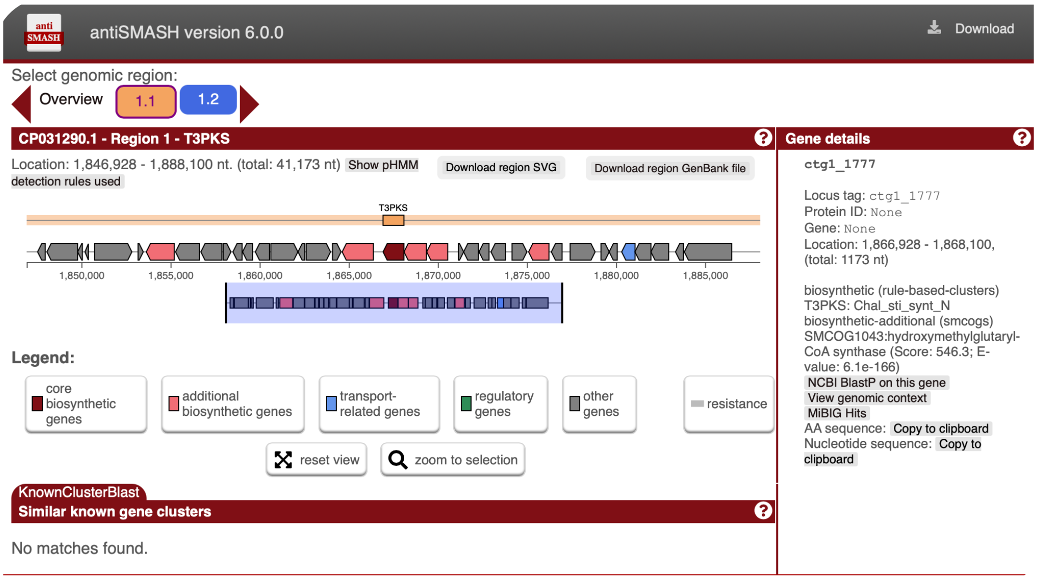

(B) gutSMASH

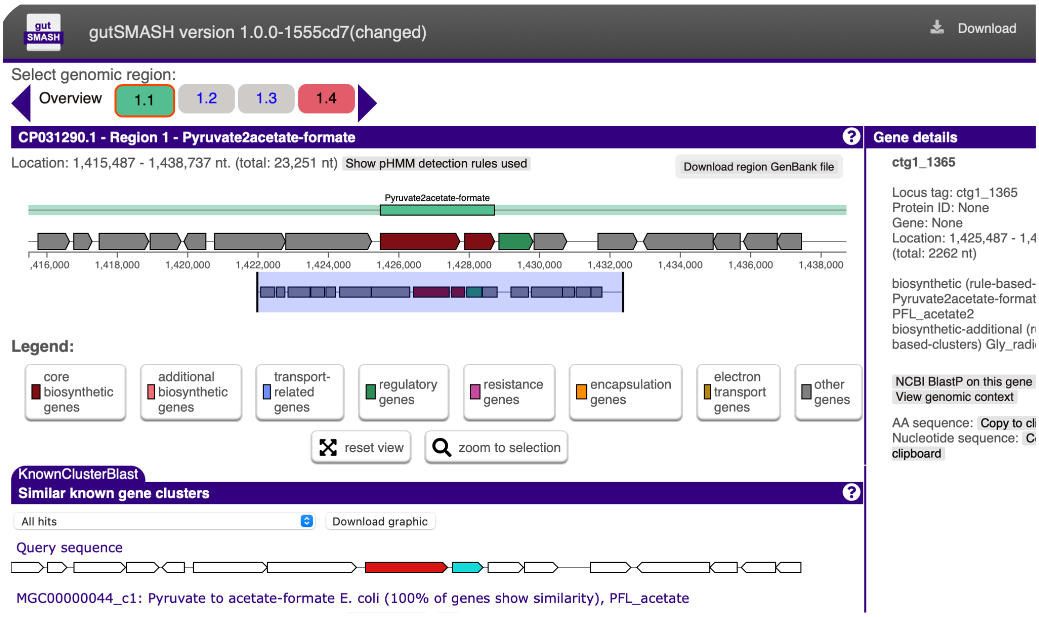

Figure S3. Analysis of *Lactobacillus rhamnosus* GG metabolic pathways

(A) Secondary metabolite gene clusters. (B) Primary metabolite gene cluster

Table S1. List of software and databases used in ProbioMinServer

Function	Software	Database
Genome identification		
Type strains comparison	Mash v2.3	NCBI prokaryote type strains (August 2023 release)
Average nucleotide identity (ANI)	https://github.com/chjp/ANI	-
Genome Circos plot	mummer2circos v1.4.2	-
	Circos v0.69-8	-
Genome annotation	eggNOG-mapper v2	eggNOG v5.0 (July 2018 release)
Safety analysis		
Antibiotic resistance genes (ARGs)	RGI v6.0.0 (BLAST homolog detection)	CARD Variants v4.0.0 (July 2022 release)
	ResFinder v4.0	resfinder_db (May 2022 release)
	AMRFinderPlus v3.10.42	(October 2022 release)
Virulence factors (VFs)	BLASTN v2.8.1+	VFDB (June 2022 release)
	VirulenceFinder v2.0.3	virulencefinder_db (December 2022 release)
Pathogenic genes (PGs)	BLASTP v2.8.1+	PHI-base v4.14 (November 2022 release)
Plasmid genes	PlasmidFinder v2.0.1	plasmidfinder_db (January 2023 release)
Prophage regions	Phigaro v2.3.0	(August 2017 release)
Functional analysis		
Secondary metabolite gene clusters	antiSMASH v6.0.0	(June 2022 release)
Primary metabolite gene clusters	gutSMASH v1.0.0	(June 2022 release)

Table S2. Database search results of *Lacticaseibacillus rhamnosus* GG (NCBI Assembly ID: GCA_003353455.1)

Rank	Genome ID	Strain[a]	Host	Allelic distance[b]	ARGs	VFs	PGs	PPRS
1	GCA_000011045.1	*ATCC 53103	-	0	0	0	2	2.00
2	GCA_003353455.1	*GG	*Homo sapiens*	0	0	0	2	2.00
3	GCA_901830405.1	AMBR1	-	0	3	0	2	2.24
4	GCA_000026505.1	*GG (ATCC 53103)	-	1	0	0	2	2.00
5	GCA_002158925.1	4B15	-	1	0	0	2	2.00
6	GCA_003052965.1	DS4_11	*Homo sapiens*	1	0	0	2	2.00
7	GCA_902381635.1	MGYG-HGUT-01293	-	1	0	0	2	2.00
8	GCA_000712515.1	PEL6	-	2	0	0	2	2.00
9	GCA_002103215.1	Lrh46	*Homo sapiens*	2	0	0	2	2.00
10	GCA_004010975.1	LR-B1	*Homo sapiens*	2	0	0	2	2.00
11	GCA_004125395.1	LR-B2	*Homo sapiens*	2	0	0	2	2.00
12	GCA_009679345.1	BIOML-A5	*Homo sapiens*	2	0	0	2	2.00
13	GCA_901830355.1	AMBR6	-	2	3	0	2	2.24
14	GCA_901830425.1	AMBR5	-	2	3	0	2	2.24
15	GCA_002025085.1	WQ2	-	3	0	0	2	2.00
16	GCA_004125455.1	LR-CVC	*Homo sapiens*	3	0	0	2	2.00
17	GCA_008831425.1	BIO6870	*Homo sapiens*	3	0	0	2	2.00
18	GCA_015238575.1	JL-1	*Homo sapiens*	3	0	0	2	2.00
19	GCA_017795605.1	LDTM7511	*Homo sapiens*	3	0	0	2	2.00
20	GCA_001657055.1	Lrh18	*Homo sapiens*	4	0	0	2	2.00
21	GCA_004125475.1	LR-S	*Homo sapiens*	4	0	0	2	2.00
22	GCA_001656545.1	Lrh32	*Homo sapiens*	5	0	0	2	2.00
23	GCA_001981725.1	RI-004	-	5	0	0	2	2.00
24	GCA_000712505.1	PEL5	-	6	0	0	2	2.00
25	GCA_001044415.1	313	*Homo sapiens*	7	0	0	2	2.00
26	GCA_001657075.1	Lrh17	*Homo sapiens*	7	0	0	2	2.00
27	GCA_001657115.1	Lrh14	*Homo sapiens*	7	0	0	2	2.00
28	GCA_001656535.1	Lrh8	*Homo sapiens*	9	0	0	2	2.00
29	GCA_001656815.1	Lrh7	*Homo sapiens*	9	0	0	2	2.00
30	GCA_001756565.1	*HCT70	-	9	0	0	2	2.00

[a] Strains annotated as probiotics in the literature are indicated with an asterisk.
[b] Allelic distance is calculated with cgMLST analysis.
